# Multiplex PCR for Detection of Herpes Simplex Viruses Type-1 and Type-2, Cytomegalovirus, Varicella-zoster Virus, and Adenovirus in Ocular Viral Infections

**DOI:** 10.18502/jovr.v16i1.8243

**Published:** 2021-01-20

**Authors:** Ahmed Nishat H, Satpathy Gita, Rohan Chawla, Radhika Tandon

**Affiliations:** ^1^Ocular Microbiology, Dr. R P Centre, All India Institute of Medical Sciences, New Delhi, India; ^2^Ophthalmology, Dr. R P Centre, All India Institute of Medical Sciences, New Delhi, India

**Keywords:** Adenovirus, Cytomegalovirus, Herpes Simplex Virus, Multiplex Polymerase Chain Reaction, Varicella-zoster Virus

## Abstract

**Purpose:**

Most common viruses causing ocular infections are Herpes Simplex Viruses (HSV) type 1 and type 2, Cytomegalovirus (CMV), Varicella-zoster Virus (VZV), and few strains of Adenovirus. Diagnosis of these infections through clinical manifestations and using conventional methods has a number of limitations. The purpose of this study was to develop a multiplex Polymerase Chain Reaction (PCR) for simultaneous detection of all pathogenic viruses from ocular infections.

**Methods:**

Ten uniplex PCRs were standardized, two each for HSV type 1 (HSV-1) and type 2 (HSV-2), CMV, VZV, and Adenovirus. Various multiplexing combinations of above PCRs were put to finalize targets and reaction conditions enabling diagnosis of all in a single reaction. The uniplex and multiplex PCRs were run for known positive and negative controls, and samples from clinically suspected patients and healthy controls.

**Results:**

Out of the 170 samples from suspected ocular infections, 24.7% were positive by uniplex PCR and 22.9% were correctly identified by multiplex PCR. None of the samples negative by uniplex PCRs was positive by the multiplex PCR. The sensitivity and specificity of multiplex PCR compared to the commonly used uniplex PCRs as gold standard was 92.86% and 100%, respectively. The prevalence of different viral pathogens was 13.5% for HSV-1, followed by 5.9% for Adenovirus, 2.4% for VZV, 1.8% for HSV-2, and 1.2% for CMV.

**Conclusion:**

The establishment of multiplex PCR has found immediate application in diagnosing ocular viral pathogens in a single reaction, thus saving time, manpower, and resources by fivefold.

##  INTRODUCTION

It is estimated that by the year 2020, the blind population in India will grow to 15 million and ocular infections will account for 15% of the total burden.^[1]^ Viruses can cause a variety of ocular infections including conjunctivitis, keratitis, keratoconjunctivitis, uveitis, chorioretinitis, iridocyclitis, and acute retinal necrosis syndrome.^[2,3,4,5,6,7]^ Unattended/late treated ocular infections especially with members of *Herpesviridae *family, including Herpes Simplex Virus-1 (HSV-1), Herpes Simplex Virus-2 (HSV-2), Cytomegalovirus (CMV), Varicella-zoster Virus (VZV), and few serotypes (1–4, 7, 8, 11, 19, 37, 53, and 54) of Adenovirus can lead to loss of vision.^[8,9]^ It is often challenging to determine the causative agent, as there could be significant overlap between the clinical features especially in the early stages of the disease leading to misdiagnosis. There is therefore a need to establish a prompt diagnostic testing that is both rapid and sensitive for an early detection and to determine the choice of treatment.^[4]^ The conventional methods used for diagnosing ocular viral infections are either less sensitive and/or specific, require sophisticated equipment and infrastructure and/or explicit expertise, or have a long turnaround time. In recent decades, focus has been on molecular diagnostics for such infections, in which Polymerase Chain Reaction (PCR) has proven to be a valuable technique. In this technique, the target gene of interest, called nucleic acid template, is amplified in a thermo-cycling reaction. From a single template, billions of copies are produced, which can then be identified by post-amplification analysis. It overcomes the lower sensitivity of conventional laboratory techniques while maintaining specificity. In addition, it can also be performed on limited patient-derived ocular specimen and is less time consuming, inexpensive, and rapid.^[2]^

Multiplex PCR is a variant in which all viruses in the differential can be diagnosed in a single PCR, thus saving time and cost. The technique works on the principle that different pairs of primers are unique to different infectious agents and their amplicon size varies in length so that the visual difference is observed when PCR reaction product is resolved on an agarose gel. It has an enormous clinical value as it allows simultaneous detection of multiple target organisms in a single reaction; thus, it is more informative and requires very less starting patient specimen. Further, there are various technical advantages of using multiplex PCR including rapid diagnosis, less cumbersome procedure, cost effectiveness, and less time taken to obtain results than conventional diagnostic methods. It has increased accuracy of data normalization and is subject to fewer human pipetting errors.^[10]^

Standardization and establishment of multiplex PCR for the diagnosis of ocular viral infection has immense clinical and technical advantage. Hence, this study was planned to standardize and establish a multiplex PCR targeting all common ocular viral pathogens for accurate and rapid laboratory confirmation, thereby aiding in the implementation of correct and timely treatment and to determine the prevalence of different viruses as ocular pathogens in our patient cohort.

##  METHODS

### Ethics

Approval of the Institute Ethics Committee was taken, and the procedures were done in accordance with the Helsinki Declaration of 1975, as revised in 2000. Written informed consent was obtained from all patients and controls.

### Study Design

The current prospective case–control study was conducted over a duration of 21 months (July 2016 to March 2018) in the Ocular Microbiology section of an apex healthcare institute of North India, which caters to the tertiary healthcare requirements of Delhi and nearby six to seven states.

### Selection and Description of Participants

A total of 170 samples, clinically guided to undertake laboratory diagnostic tests for viral infections, were included as test samples. The patients had various clinical manifestations. Vitreous and aqueous aspirates were collected in sterile Eppendorf tubes; and samples like scrapings, tears, swabs, etc. were collected in sterile Eppendorf tubes containing 1 ml of phosphate buffered saline (PBS, pH 7.2). Maximum numbers of samples received were corneal scrapings (54), conjunctival swabs (45), and tears (33). Fifty tear samples from individuals devoid of any clinical symptoms of ocular infections were included as controls.

### DNA Extraction

DNA extraction was done using the QIAamp DNA extraction kit (QIAGEN, Qiagen Str. 1, 40724 Hilden, Germany), strictly following the manufacturer's instructions. As the quantity of ocular specimens was very little, elution of DNA was done in 60 µl of elution buffer. Extracted DNA samples were stored at 0–4°C until processed.

### Standardization of Multiplex PCR

Published primers unique and highly specific for HSV-1, HSV-2, CMV, VZV, and Adenovirus were used for standardizing uniplex PCRs. A total of 10 uniplex PCRs were standardized, two each for HSV-1, HSV-2, CMV, VZV, and Adenovirus.^[11,12,13]^ Results of uniplex PCRs were checked in control strains of all viruses, non-ocular stored clinical specimens positive for different study viruses, and also in extracted DNA from cultures of *Staphylococcus*, *Pseudomonas aeruginosa*, *Acanthamoeba species*, *Aspergillus flavus*, and *Fusarium species*.

The uniplex PCRs were then run at different annealing temperatures and with some variations of cycle conditions to obtain the annealing temperature and cycle conditions suitable for all five study viruses. Various multiplexing combinations of above PCRs were put to make it possible to diagnose all the above five viruses in a single reaction. The five targets, annealing temperature, and reaction conditions which were giving best results for all viruses in a single reaction were finalized. The multiplex reactions were run with control strains of all viruses, non-ocular clinical specimens positive for different study viruses, and DNA extracted from the cultures of *Staphylococcus*, *P. aeruginosa*, *A. species*, *A. flavus,* and *F. species*. All the results of uniplex and multiplex PCRs were finally verified in stored DNA of ocular specimens with known results.

Subsequently, for all clinical samples and controls, five uniplex PCRs (one for each virus) and one multiplex was run. DNA of ATCC-VR-539D, HSV-1 strain McIntyre; ATCC-VR-734D, HSV-2 strain G; OKA vaccine strain of VZV; and pooled extracted DNA positive for CMV and Adenovirus from clinical samples were used as positive controls in each run. Autoclaved MilliQ water controls were used as negative controls in each run. ATCC strains were purchased through LGC Promochem India Private Limited, Bangalore, India.

The details of primers for the five selected targets for the multiplex PCR are shown in Table 1. Both the uniplex and multiplex PCR amplification reactions were conducted in 25 µl volumes. The reaction mixture consisted of dNTPs (200 mM) – 0.5 µl, 10X buffer – 2 µl, MgCl2 – 1.2 µl, forward primer – 1 µl, reverse primer – 1 µl, Taq DNA polymerase 1U – 0.2 µl, test DNA – 2 µl, and autoclaved MilliQ water – 12.1 µl made up to 25 µl. The amplification profile chosen was as follows: initial denaturation at 95°C for 5 min, followed by 40 cycles of denaturation at 95°C for 30 sec, annealing at 55°C for 35 sec, extension at 72°C for 40 sec, and final extension at 72°C for 10 min.

**Table 1:** Details of primers for the five selected targets for the multiplex PCR


**Name **	**Primers **	**Region **	**Sequence (5'-3') **	**Size **
HSV 1	HSV 1-F	RL-2	TGGGACACATGCCTTCTTGG	147 bp
	HSV 1-R	RL-2	ACCCTTAGTCAGACTCTGTTACTTACCC	
HSV 2	HSV 2-F	gp-D	GTCGGTGTGGTGTTCGGTCATAAGCT	276 bp
	HSV 2-R	gp-D	GGCTGAATCTGGTAAACACGCTTC	
CMV	CMV-F	pol and gp-B	CACGGCCGCCACCAAGGT	392 bp
	CMV-R	pol and gp-B	AGTGGTTGGGCAGGATAAA	
VZV	VZV-F	gp	ATCGCGGCTTGTTGTTTGTCTAAT	355 bp
	VZV-R	gp	GGGCGAAATGTAGGATATAAAGGA	
Adenovirus	Adenovirus-F	Hexon	GCCGCAGTGGTCTTACATGCACATC	308 bp
	Adenovirus-R	Hexon	CAGCACGCCGCGGATGTCAAAGT	
RL, long repeat region; gp, glycoprotein region; pol, DNA polymerase gene				

**Table 2:** Results of uniplex and multiplex PCRs in test samples


**Pathogenic virus **	**Numbers identified by uniplex PCR **	**Numbers identified by multiplex PCR **
HSV-1	23	22
HSV-2	03	03
CMV	02	02
VZV	04	03
Adenovirus	10	09
** Total Positive **	**42 **	**39 **
** Negative **	**128 **	**131 **

**Table 3:** Results of uniplex and multiplex PCRs from patients with different clinical diagnosis


**Clinical diagnosis**	**Number of specimens received**	**Positive by uniplex PCR**	**Positive by multiplex PCR**
Conjunctivitis	41	17	16
Keratitis	81	12	11
Blepharitis	3	0	0
Lid and periocular vesicles	5	4	3
Uveitis	5	2	2
Chorioretinitis	5	2	2
Endophthalmitis	3	0	0
Others	27	5	5
**Total**	**170**	**42**	**39**

### Electrophoresis and Documentation

**Figure 1:**
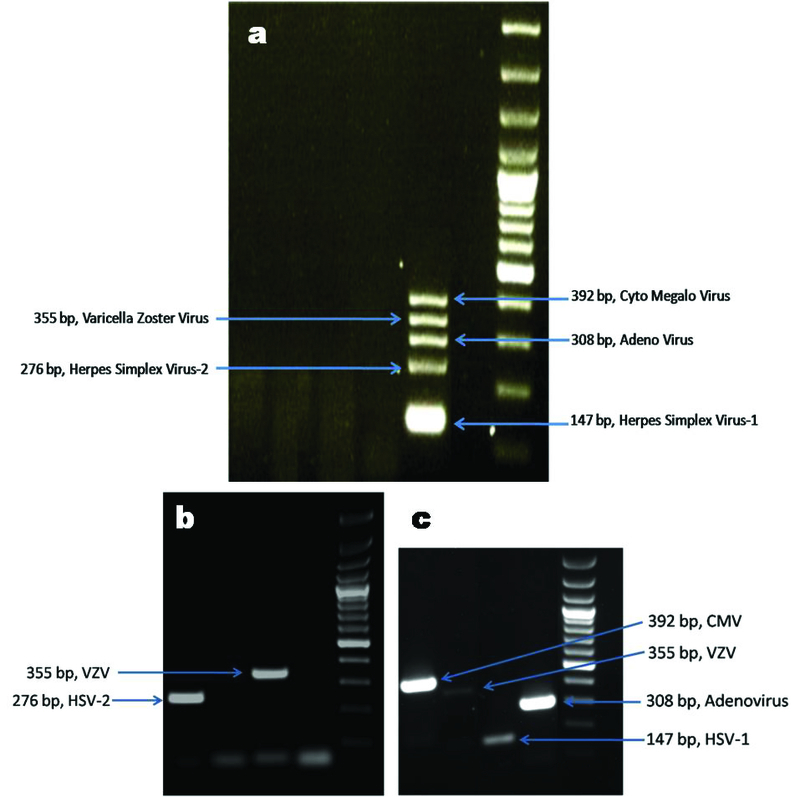
Gel images of positive controls and positive test samples by multiplex PCR. (a) Multiplex PCR simultaneously detecting all five viruses, (b) Ocular specimens positive for HSV-2 and VZV by multiplex PCR, and (c) Ocular specimens positive for CMV, VZV, HSV-1, and Adenovirus by multiplex PCR.

Following the PCR, the amplicons were resolved on a 1.5% agarose gel. Visualization was done with the aid of ethidium bromide (0.5 µg/ml) under ultraviolet illumination using gel documentation system – Biospectrum R 810 Imaging System – UVP (2066 W. 11th St., Upland, CA 91786, USA). Figures 1a, 1b, and 1c show the gel images of positive controls and positive test samples by multiplex PCR.

**Figure 2:**
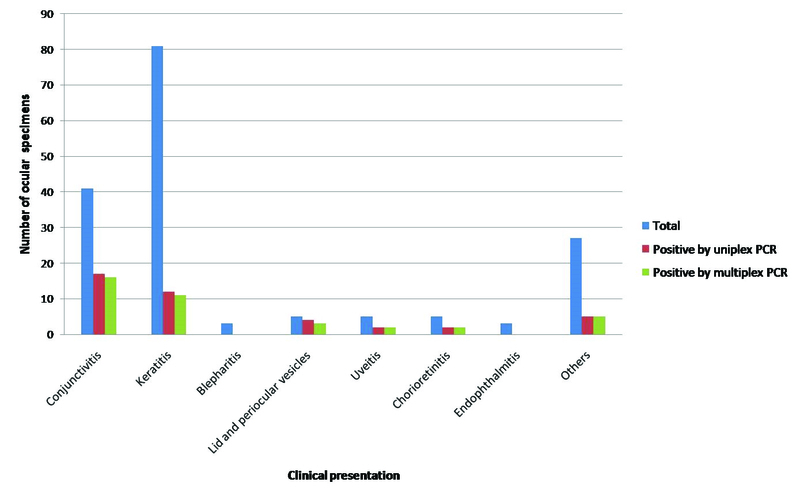
Distribution of specimens received and results of uniplex and multiplex PCRs from patients with different clinical diagnosis.

**Figure 3:**
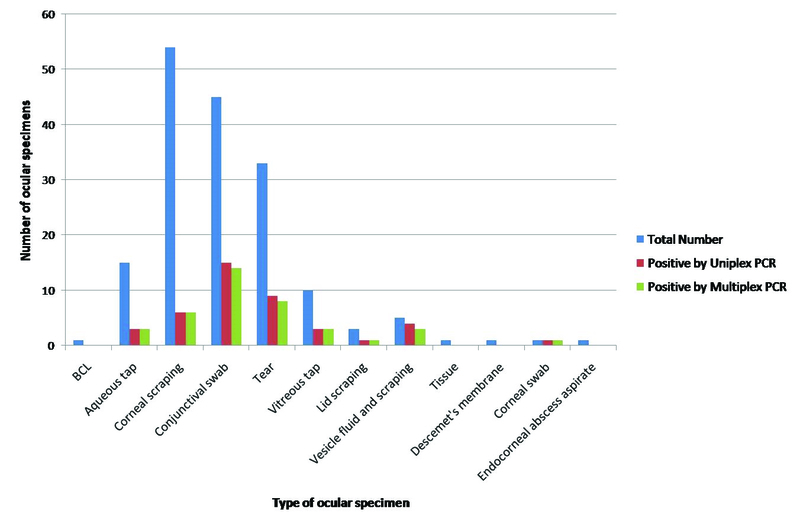
Sample-wise distribution of results of uniplex and multiplex PCRs.

Results of uniplex and multiplex PCRs were entered in Microsoft Excel sheets (Microsoft${}^{R}$ Office Excel${}^{R}$ 2007 [12.0.4518.1014] MSO [12.0.4518.1014]). Sensitivity, specificity, positive and negative predictive values, and accuracy of multiplex PCR was calculated with uniplex PCRs as gold standard.

##  RESULTS

### Controls

Both uniplex and multiplex PCRs were correctly able to identify the viruses from stored DNA of positive ocular and non-ocular samples. None of the DNA from non-viral ocular pathogens – *Staphylococcus*, *P. aeruginosa*, *A. species*, *A. flavus* and *F. species* – was positive for any of the viruses by uniplex or multiplex PCR. None of the 50 control samples from healthy eyes were positive for any of the viruses by uniplex or multiplex PCR.

### Clinical Samples

Over the duration of 21 months, 170 specimens from clinically suspected ocular viral infections were received. Uniplex and multiplex PCRs for HSV-1 and HSV-2, CMV, VZV, and Adenovirus were performed for all patients' specimens.

Table 2 shows the results of uniplex and multiplex PCRs in test samples. Out of the 170 samples from cases of suspected ocular infections, 42 (24.7%) were positive for some of the five viruses tested by uniplex PCR. Multiplex PCR was able to correctly detect 39 out of 42 positives of uniplex PCRs (22.9% of 170). None of the samples were positive for more than one virus. None of the samples negative by uniplex PCRs was positive by the multiplex PCR. Thus, the sensitivity, specificity, positive predictive value, negative predictive value, and accuracy of the multiplex PCR was 92.86%, 100%, 100%, 97.71%, and 98.24%, respectively.

The prevalence of different viral pathogens causing ocular infections as determined by the PCR was found to be 13.5% for HSV-1 (23 out of 170 cases positive), followed by 5.9% (10 positive) for Adenovirus, 2.4% (four positive) for VZV, 1.8% (three positive) for HSV-2, and 1.2% (two positive) for CMV (Table 2).

Table 3 and Figure 2 show the distribution of samples received from patients with different clinical diagnoses. Maximum samples were from patients having keratitis, followed by conjunctivitis. Viral infections could be diagnosed using multiplex PCR in 60% of patients having lid and periocular vesicles. Viral etiology could also be clinched in 39% of conjunctivitis and 40% each of uveitis and chorioretinitis patients using the multiplex PCR.

Maximum positivity was observed in vesicle fluid and scrapings (60%), followed by lid scrapings (33.3%), conjunctival swabs (31.1%), and vitreous tap (30%). The positivity in tear samples was found to be 24.2% (Figure 3).

##  DISCUSSION

Ocular viral infections can range from simple self-limiting discomfort to possibly vision challenging manifestations. The clinical manifestations of such infections are not specific for a particular virus; frequently, the differential includes a number of viruses. There often is an overlap of signs and symptoms with non-viral infections and some non-infective conditions. This is more common in tertiary care centers where the patients often come after partial treatment performed outside, have some underlying immune-compromise, or have some complications of the infection. Most common viruses causing ocular infections are HSV-1, HSV-2, CMV, VZV, and Adenovirus, which are difficult to differentiate by clinical findings alone; nevertheless, the differentiation is important as it determines the choice of treatment.^[2]^ Late or inappropriate treatment of such infections due to delayed diagnosis can compromise the vision of the patient. PCR is now a popular diagnostic test for viral infections; however, its application is limited in clinical situations where the differential diagnosis takes account of several pathogens. Running a PCR for each pathogen in the differential is a time- and resource-consuming process; moreover, each extra reaction has its own share of errors; and sometimes it is not possible to do many reactions as the specimen size is minute in ocular infections. There is a dearth of an accurate, rapid, and cost-effective diagnostic test which can be undertaken on limited ophthalmic sample volume.

A multiplex PCR was thus standardized targeting HSV-1, HSV-2, CMV, VZV, and Adenovirus. This enabled the diagnosis of all five common ocular viral pathogens in a single reaction. The sensitivity and specificity of the multiplex PCR was found to be 92.5% and 100%, respectively. The multiplex PCR was made sensitive for diagnosing ocular viral infections by multiplexing the five most common pathogenic viruses. Specificity was established by simultaneously using uniplex PCRs in all clinical specimens and controls; also, the multiplex PCR did not show any false positivity in DNA from non-viral pathogens. The multiplex PCR was found to be useful in tear samples, which are the least invasive of ocular specimens, showing viral detection in 24.2% of tear samples received. Furthermore, the test was able to clinch diagnosis in 40% of uveitis and chorioretinitis cases, establishing the etiology of which is otherwise very difficult and time-consuming.

In the current study, we also looked for the prevalence of different viruses as ocular pathogens in our patient cohort, and for any asymptomatic carriage in control group.

The overall positivity for viral pathogens in clinically suspected ocular viral infections as detected by uniplex PCR was 24.7% with HSV-1 being the most common (14.4%), followed by Adenovirus (5.9%). None of the healthy controls were positive for any of the viruses.

Our results are comparable to similar studies done in India and abroad.

A study from Japan utilizing multiplex PCR for the detection of HSV-1, HSV-2, CMV, and VZV in different ocular infections has reported the multiplex PCR to be as good as uniplex PCRs for each of the viruses. They have reported a positivity of 57.6% in clinically suspected ocular viral infection cases. Amongst the positives, HSV-1 was the most common (68.4%), followed by VZV (31.6%).^[13]^ Sugitha *et al* from Japan have recently described the results of utilization of a multiplex PCR for the detection of eight herpes viruses and the parasite *Toxoplasma gondii* in cases with uveitis and endophthalmitis. They reported sensitivity and specificity of 91.3% and 98.8%, respectively. The positivity in their study group was 34%, with CMV and VZV being the most common viral pathogens.^[14]^

Elnifro *et al* in their study in United Kingdom tested a multiplex PCR for detecting HSV, Adenoviruses, and *Chlamydia* in eye swabs. Although they observed a 10-fold fall in the sensitivity of detection limit using multiplex PCR, there was no significant difference in the diagnostic sensitivity of multiplex PCR when compared to that of individual uniplex PCRs.^[15]^

Another similar study from India reported multiplex PCR for HSV, VZV, and CMV in ocular specimens. The authors have not compared their results with uniplex PCRs in all samples; however, they have established detection limits of multiplex PCR using several dilutions of standard strains. The sensitivity of uniplex PCR for HSV, VZV, and CMV was 4, 4, and 6 PFU/ml, respectively, while that of multiplex PCR was 4, 4, and 12 PFU/ml, respectively. The authors have also established specificity using diverse DNA samples derived from non-viral infections and non-infectious conditions of the eyes. The most common viral infections found were HSV (83.6%), followed by VZV (2%) and CMV (1.4%).^[12]^

The most recent study is from United States, in which Bizpo *et al* have reported results of a qualitative multiplex real-time PCR for the identification of common pathogens causing uveitis. They have targeted viruses HSV-1, HSV-2, VZV, CMV and parasite *T. gondii* and have found the multiplex real-time PCR to be highly specific with a limit of detection of 20 genome copies for viral pathogens and 200 genome copies for *T. gondii*.^[16]^

There are a few limitations of the study. We have not found the detection limit in terms of genome copy number of uniplex and multiplex PCR for each virus, which would be a better marker of sensitivity of the test. Also, involvement of samples from other ophthalmic centers and inclusion of bioinformatics and in silico analysis would have added weight to the results of the present study.

To conclude, the present study has shown that the multiplex PCR targeting five common viral infections can serve as a valuable diagnostic tool for ophthalmic viral infections. It reduces the turnaround time to diagnose specific viruses, and also the chances of errors associated with putting multiple reactions; at the same time saving on hands on work and cost of diagnosis. The study has also thrown light on current epidemiology of ocular viral infections.

The understanding of current pattern of ocular viral infections and utilization of multiplex PCR for diagnosis can go a long way in improving the management of patients having viral infections of the eyes.

##  Financial Support and Sponsorship

The study was funded by the 'Institutional Research Grant' from All India Institute of Medical Sciences, New Delhi, India.

##  Conflicts of Interest

There are no conflicts of interest.
